# Cervical lymph node metastases from thyroid cancer: does thyroglobulin and calcitonin measurement in fine needle aspirates improve the diagnostic value of cytology?

**DOI:** 10.1186/1472-6890-13-7

**Published:** 2013-02-19

**Authors:** Enke Baldini, Salvatore Sorrenti, Cira Di Gioia, Corrado De Vito, Alessandro Antonelli, Lucio Gnessi, Giovanni Carbotta, Eleonora D’Armiento, Paolo Miccoli, Enrico De Antoni, Salvatore Ulisse

**Affiliations:** 1 Department of Experimental Medicine, University of Rome, Rome, Italy; 2 Department of Surgical Sciences, University of Rome, Rome, Italy; 3 Department of Radiological, Oncological and Anato-Pathological Sciences, University of Rome, Rome, Italy; 4 Department of Public Health and Infectious Diseases, “Sapienza” University of Rome, Rome, Italy; 5 Department of Internal Medicine, University of Pisa, Pisa, Italy; 6 Department of Surgery, University of Pisa, Pisa, Italy; 7 Department of Experimental Medicine, “Sapienza”, University of Rome, Viale Regina Elena, 324, 00161, Rome, Italy

**Keywords:** Thyroid cancer, Lymph node metastasis, Diagnosis, Thyroglobulin, Calcitonin, Fine-needle aspiration cytology, Follow-up

## Abstract

**Background:**

Measurement of thyroglobulin (Tg) protein in the washout of the needle used for fine needle aspiration biopsy cytology (FNAB-C) has been shown to increase the sensitivity of FNAB-C in identifying cervical lymph node (CLN) metastasis from well-differentiated thyroid cancer (TC). In this study, we evaluated whether routine measurement of Tg protein (FNAB-Tgp), Tg mRNA (FNAB-Tgm) and calcitonin (CT) mRNA (FNAB-CTm) in the FNAB washout of CLN increases the accuracy of FNAB-C in the diagnosis of suspicious metastatic CLN.

**Methods:**

In this prospective study 35 CLN from 28 patients were examined. Histology showed metastatic papillary TC (PTC) in 26 CLN, metastatic medullary TC (MTC) in 3 CLN, metastatic anaplastic TC (ATC) in 3 CLN and 3 metastatic CLN from extra-thyroidal cancers.

**Results:**

The overall accuracy of FNAB-C was 84.4%, reaching 95.7% when the analysis was restricted to PTC. Both FNAB-Tgp and FNAB-Tgm compared favorably with FNAB-C and shown diagnostic performances not statistically different from that of FNAB-C. However, FNAB-Tgp and FNAB-Tgm/FNAB-CTm were found useful in cases in which cytology results were inadequate or provided diagnosis inconsistent with patient's clinical parameters.

**Conclusions:**

We demonstrated that FNAB-C, Tg/CT mRNA and Tg protein determination in the fine-needle washout showed similar accuracy in the diagnosis of metastatic CLN from TC. The results of this study suggest that samples for Tg protein and Tg/CT mRNA measurements from CLN suspicious for metastatic TC should be collected, but their measurements should be restricted to cases in which FNAB-C provides uninformative or inconsistent diagnosis with respect to patient's clinical parameters.

## Background

Thyroid cancer represents the most frequent endocrine neoplasia, accounting for 1.7% (0.7% in male and 2.7% in female) of all new malignant diseases and 0.5% (0.3% in male and 0.7% in female) of deaths related to cancer worldwide [1,2]. Thyroid carcinomas originate mostly from the epithelial follicular cells and are represented for 95% by the differentiated (DTC) papillary (PTC) and follicular (FTC) carcinomas, while 1-2% of them are the undifferentiated and unvaryingly fatal anaplastic carcinomas (ATC) [3]. The remaining 3-4% are medullary thyroid carcinomas (MTC) derived from the parafollicular C cells [4]. The accurate diagnosis of locoregional lymph node metastasis is of primary importance for the initial surgical approach as well as for prognostic stratification and follow-up [4-9]. In this context, fine-needle aspiration biopsy cytology (FNAB-C) represents the gold standard technique for the detection of cervical lymph node (CLN) metastasis [4-9]. The latter, however, relies on the experience and ability of the cytopathologist, and may be a challenging diagnostic category as CLN could harbor metastasis from a multiplicity of extrathyroidal malignancies or be affected by several non-tumoral diseases [9-11]. In addition, inadequate cellularity or nonrepresentative sampling, often associated with cystic lymph nodes, prevents diagnosis in about 20% of specimens [12-14]. Over the last two decades, a number of studies have demonstrated that measurement of thyroglobulin protein (Tgp) in the washout of the needle used for FNAB (FNAB-Tgp) increases the sensitivity of FNAB-C in identifying CLN metastasis from DTC [14-27]. As a consequence, routine association of FNAB-Tgp with FNAB-C in the diagnosis of CLN metastasis from papillary and follicular thyroid cancers has been recommended [6,7,15]. Similarly, it has been shown that lymph node detection of Tg mRNA (Tgm) in fine-needle washout implemented the FNAB-C sensitivity for the diagnosis of metastatic CLN from DTC, even if this still needs to be validated on larger case-studies [28].

In the present work we evaluated, in 35 consecutive CLN for which the histological diagnosis was available, if routine measurement of thyroglobulin (Tg) protein and Tg and calcitonin (CT) mRNA in the washout of the needle used for FNAB increases the sensitivity of FNAB-C in identifying cervical lymph node (CLN) metastasis from either DTC or MTC. The results obtained suggest that while samples for Tg protein and Tg/CT mRNA measurements from CLN suspicious for metastatic thyroid cancer should be always collected, their measurements should be restricted to cases in which FNAB-C gives uninformative or inconsistent diagnosis with respect to patient’s biochemical and/or clinical parameters.

## Methods

### Patients

The case study included 35 cervical lymph nodes (CLN) with definitive diagnosis from 28 consecutive patients referred, from September 2004 to October 2012, to the outpatients’ clinic of Endocrinology and Thyroid Diseases of the Policlinico Umberto I general hospital of Rome (Italy). The study was approved by the ethical committee of the Policlinico Umberto I hospital in agreement to the declaration of Helsinki and all patients gave written informed consent. Patients, 7 males and 21 females with a median age of 39.5 yr (range 20-72 yr), with single or multiple suspicious CLN underwent fine-needle aspiration biopsy (FNAB) followed by cytological (FNAB-C) evaluation, Tgp measurement, and Tgm and CTm assessment in the fine needle washout. Among them, 9 patients previously underwent total thyroidectomy with histological diagnosis of papillary thyroid cancer (see Table 1). All patients with thyroid cancer underwent unilateral neck dissection. The histological diagnosis showed metastatic PTC in 21 patients, metastatic MTC in 2, metastatic ATC in 2 and metastasis from extrathyroidal cancers in the remaining 3 patients (1 non-Hodgkin lymphoma, 1 rhino-pharyngeal and 1 lung carcinoma). The enlarged lymph nodes from the latter 3 patients were evaluated since they all presented thyroid nodules with ultrasound features suspicious of malignancy (i.e. hypoechogenicity, presence of irregular margins and microcalcifications).

**Table 1:** Cytological, molecular and histological diagnoses of suspicious metastatic cervical lymph nodes (CLN) from thyroid cancer patients

**CLN number**	**FNAB-C**	**FNAB-Tgp (ng/FNAB)**	**FNAB-Tgm**	**FNAB-CTm**	**Histology**
**1**	IN	6620	+	-	Met. PTC
**2**	IN	7480	+	-	Met. PTC
**3**	Met. PTC	5652	+	-	Met. PTC
4*	Met. PTC	5293	+	-	Met. PTC
5*	Met. PTC	18612	+	-	Met. PTC
**6**	Met. PTC	1625	+	-	Met. PTC
**7**	Met. MTC	0.9	-	+	Met. MTC
8*	IN	37.50	IN	IN	Met. PTC
**9**	Met. PTC	26108	+	-	Met. PTC
**10**	Met. ATC	UND	+	-	Met. ATC
**11**	Met. PTC	37250	ND	ND	Met. PTC
**12**	Met. PTC	0.8	+	-	Met. ATC
**13**	Met. PTC	2.82	-	-	Met. ATC
14*	Met. PTC	114	+	-	Met. PTC
15*	Met. PTC	288	-	-	Met. PTC
**16**	Met. PTC	1151	+	-	Met. PTC
**17**	Met. PTC	3714	+	-	Met. PTC
**18**	Met. PTC	480	+	-	Met. PTC
**19**	Met. PTC	UND	-	+	Met. MTC
**20**	Met. PTC	UND	-	+	Met. MTC
21*	Met. PTC	62908	IN	IN	Met. PTC
**22**	Met. PTC	72.3	+	-	Met. PTC
**23**	Met. PTC	10349	+	-	Met. PTC
**24**	Met. PTC	384	ND	ND	Met. PTC
25*	Met. PTC	728	ND	ND	Met. PTC
**26**	Met. PTC	53	+	-	Met. PTC
**27**	Met. PTC	ND	+	-	Met. PTC
28*	Met. PTC	ND	+	-	Met. PTC
29*	Met. PTC	ND	+	-	Met. PTC
**30**	Epithelial CA	UND	-	-	Met. RFC
**31**	Lymphoma	10.9	-	-	Lymphoma
**32**	Met. LC	UND	-	-	Met. LC
**33**	Met. PTC	3361	+	-	Met. PTC
34*	Benign	4395	-	-	Met. PTC
35*	Met. PTC	4388	+	-	Met. PTC

FNAB-C, fine-needle aspiration biopsy (FNAB) cytology; FNAB-Tgp, FNAB thyroglobulin protein; FNAB-Tgm, FNAB Tg mRNA; FNAB-CTm, FNAB CT mRNA; ND, non-determined; UND, undetectable; IN, inadequate; Pos. WBS, positive whole body scan; RFC, rhino-pharyngeal carcinoma; CA, cancer; LC, lung cancer. * CLN from previously thyroidectomized patients.

### Ultrasonography, fine-needle aspiration biopsy (FNAB) and fine-needle washout

Thyroid ultrasonography (US) was performed on the cervical area using the Aplio XV (Toshiba, Japan) system equipped with a linear transducer (PLT-805AT). The scanning was performed on patients’ neck hyperextended in supine position. US parameters suggestive of malignant lymph node infiltration included: rounded shape and long-to-short axis ratio inferior to 1.5; irregular echogenicity; presence of microcalcifications; irregular vascularity, i.e. peripheral or mixed (hilar and peripheral) vascularity. Following US, patients selected for the FNAB were instructed not to take aspirin or any other anticoagulant in the 5 days prior to biopsy. A 25 -gauge needle, attached to 20 ml plastic syringe, was used to aspirate nodes under US assistance. Two to three separate aspiration from each CLN were made. All aspirates were smeared directly on glass slides for cytological examination as described below. The needle was then washed with 1 ml of phosphate buffered saline (PBS) by multiple pumping actions and the suspension was centrifuged at 1200 rpm for 5 min. The supernatant was collected and frozen to -20°C until analyzed for Tg protein level (FNAB-Tgp), while the pellet was quickly frozen in liquid nitrogen and stored at -80°C.

### Cytological and histological analysis

Following FNAB the needle’s material was expelled onto glass slides and smeared with a second slide to spread the material across the surface. The slides were then either air-dried or wet-fixed using the Bio-Fix (Bio-Optica, Milan, Italy). Air-dried slides were stained with a May Grunwald-Giemsa solution, while the wet-fixed slides were stained with the Papanicolaou solution. For the histological diagnosis, soon after lymphadenectomy the tissue was placed in 10% buffered formalin and paraffin embedded. Four μm thick sections were then prepared and stained with hematoxylin and eosin [29]. Both cytological and histological diagnoses were made by two expert pathologists and were concordant in all cases. Both pathologists were, at the time of their diagnoses, unaware of Tgp, Tgm and CTm results as well the molecular biologists were unaware, at the time of their diagnoses, of the patologists diagnoses.

### Tg protein and Tg autoantibody measurements

Tgp levels in the FNAB washout were measured using the immunoluminometric assay (ILMA) Tg-PluS (B.R.A.H.M.S., Hennigsdorf, Germany). The analytical and functional sensitivity of the assay was, respectively, 0.02 ng/ml and 0.15 ng/ml. Tg values below the functional sensitivity were considered undetectable [30]. Samples were assayed in duplicate either non-diluted or following 1:10, 1:100 and 1:1000 dilutions in PBS. Results were expressed as ng/FNAB [19]. Even if FNAB-Tgp measurement has been reported to be non-affected by serum Tg autoantibodies [31], all FNAB washout samples were routinely assayed for Tg autoantibodies using the Anti-Tgn kit from B.R.A.H.M.S. with a functional sensitivity of 20 U/ml. In all samples Tg autoantibodies were undetectable (below 20 U/ml).

### Extraction and analysis of RNA

The above mentioned pellets have been resuspended in 500 μl of Isol-RNA lysis reagent (Eppendorf, Milano, Italy) and total RNA extracted as previously described [32]. In particular, the precipitation of RNA has been maximized by adding 20 μg of molecular biology-grade glycogen to isopropanol. The RNA has been reverse-transcribed using anchored Oligo(dT)23 primers and M-MLV reverse transcriptase (Sigma Aldrich Co. St-Louis, MO). Negative controls have been performed in parallel for all samples omitting the reverse transcriptase in the RT reaction mix. PCR mix has been prepared with dNTPs 0.2 mM, 1.5 U of HotMaster Taq DNA Polymerase (Eppendorf, Milano, Italy), 5 μl of 10x Taq buffer containing Mg^2+^, specific Tg, CT and beta-2-microglobulin (B2M) primers 0.5 μM, 2-3 μl of cDNA, and molecular biology-grade water to 50 μl. In particular, the sequences of the above primers were as follows: Tg, forward CTCTGGAAAGATTCTGACATGG (exon 28-29), reverse CTCTGGAAAGATTCTGACATGG (exon 30-31) (amplicon 243 bp); CT, forward CCTTCCTGGCTCTCAGCATC (exon 2), reverse GAGTTTAGTTGGCATTCTGG (exon 4) (amplicon 407); B2M, forward CAGCAGAGAATGGAAAGTC (exon 2), reverse CATGCTGCTTACATGTCTCG (exon 3) (amplicon 269 bp). After 2 min of initial denaturation at 94°C, 40 cycles have been run as follows: 94°C for 20 sec, 56-60°C for 10 sec, 65°C for 50 sec. Positive controls, represented by normal human thyroid tissue for Tg mRNA and the MTC derived cell line TT for CT mRNA, have been included each time. The obtained amplicons have been visualized by agarose gel electrophoresis, and their specificities have been checked by automated DNA sequencing (Primm, Milano, Italy).

### Statistical analysis

Sensitivity, specificity, diagnostic accuracy, positive and negative predictive value of each diagnostic test were calculated. Differences in sensitivity, specificity, negative and positive predictive values and accuracy among the different tests were evaluated by the Fisher exact test. Cohen’s k coefficient was used to estimate the agreement between various diagnostic tests. Statistical calculations were performed using Stata version 8.0 (College Station, Texas, Stata Corporation, 2003). The results were considered statistically significant when the two-tailed p value was <0.05.

## Results

### FNAB cytology (FNAB-C) versus histological diagnosis

As reported in Table 1, FNAB-C was inadequate in 3 (8.6%) out the 35 samples of CLN, 2 of which were from anaechoic (cystic) CLN and 1 from hypoechoic CLN. The FNAB-C diagnosis of the 32 CLN was correctly provided in 27 cases, with accuracy of 84.4%. In the remaining 5 cases FNAB-C diagnosis of metastatic PTC in 4 CLN was incorrect because the histology demonstrated 2 metastatic MTC (Table 1, CLN n. 19 and 20) and 2 metastatic ATC (Table 1, CLN n. 12 and 13), while the fifth CLN with a benign cytology turned out to be a metastatic PTC (Table 1, CLN n. 34) These 5 FNAB-C diagnoses were considered as false negative. The specificity, sensitivity, positive (PPV) and negative (NPV) predictive values and accuracy of FNAB-C are reported in Table 2.

**Table 2:** Diagnostic performance of different tests for diagnosis of suspicious metastatic cervical lymph nodes (CLN) from thyroid cancer patients (n = 35)

**Test**	**Sensitivity**	**Specificity**	**PPV**	**NPV**	**Accuracy**
FNAB-C	84.4 (27/32) [67.2-94.7]	N.D. (0/0) [N.D.]	100 (27/27) [87.2-100]	N.D. (0/5) [0-52.2]	84.4 (27/32) [67.2-94.7]
FNAB-Tgp	92.3 (24/26) [74.9-99.1]	83.3 (5/6) [35.9-99.6]	96.0 (24/25) [79.7-99.9]	71.4 (5/7) [29.0-96.3]	90.6 (29/32) [77.7-103.5]
FNAB-Tgm	87.5 (21/24) [67.6-97.3]	100 (6/6) [54.1-100]	100 (21/21) [83.9-100]	66.7 (6/9) [29.9-92.5]	90.0 (27/30) [76.3-103.7]
FNAB-CTm	100 (3/3) [29.2-100]	100 (27/27) [87.2-100]	100 (3/3) [29.2-100]	100 (27/27) [87.2-100]	100 (30/30) [100-100]

PPV, positive predictive value; NPV, negative predictive value; C, cytology; Tgp, thyroglobulin protein; Tgm, thyroglobulin mRNA; CTm, calcitonin mRNA. N.D., non-determined. In round brackets the number of cases. In square brackets the 95% confidence interval.

### FNAB Tg protein (FNAB-Tgp) versus histological diagnosis

**Figure 1:**
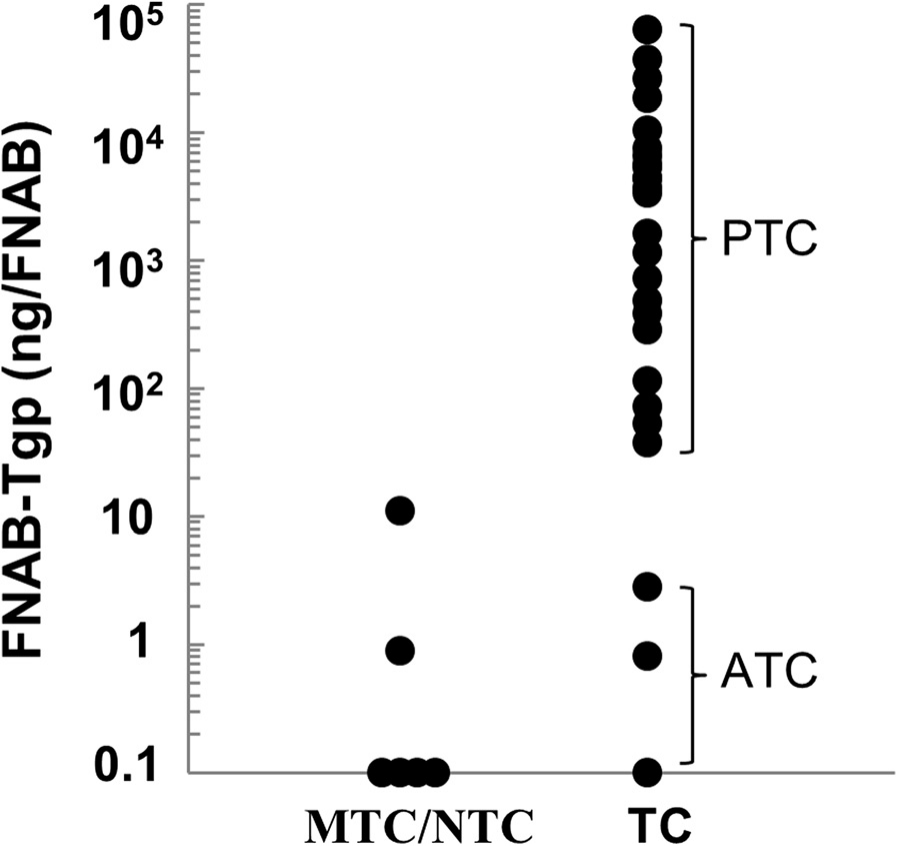
**Thyroglobulin concentrations in fine-needle biopsies of 32 cervical lymph nodes with histological diagnosis.** MTC, medullary thyroid cancer (n = 3); NTC, non-thyroidal cancers (n = 3); TC, epithelial thyroid cancer (n = 26); PTC, papillary thyroid cancer (n = 23); ATC, anaplastic thyroid cancer (n = 3).

As reported in Figure 1 and Table 1, Tgp level in the fine-needle washout was measured in 32 out of the 35 CLN with definitive diagnosis. In order to limit false negative results considered unacceptable and in agreement with other studies, we decided to adopt a Tgp cut-off value of 1 ng/FNAB [16]. FNAB-Tgp provided correct diagnoses in 29 CLN (24 true positive and 5 true negative), false negative results (Tgp level <1 ng/FNAB) in 2 harboring metastatic ATC and false positive result in one with definitive diagnosis of lymphoma. Among the 23 CLN in which histology showed metastatic PTC, Tgp values ranged from 37.5 ng to 62908 ng (mean 8742 ng/FNAB; SD 15001 ng). In Table 2, specificity, sensitivity, PPV, NPV and accuracy of FNAB-Tgp are reported.

### FNAB Tg (FNAB-Tgm) and CT (FNAB-CTm) mRNA versus histological diagnosis

Tg and CT mRNA levels were measured in 32 out of the 35 CLN with definitive diagnosis. In 2 cases samples were judged inadequate because of the absence of β2-microglobulin mRNA. As shown in Tables 1 and 2, the analysis of Tg mRNA in the fine-needle washout of the 30 samples provided a correct diagnosis in 27 cases (21 true positive and 6 true negative CLN) and 3 false negative results, 1 with definitive diagnosis of metastatic ATC and 2 of metastatic PTC.

The CT mRNA analysis provided correct results in all 30 samples (3 true positive and 27 true negative).

### Comparison of the different tests in the diagnosis of suspicious CLN

The comparison of the diagnostic parameters of FNAB-C, FNAB-Tgp and FNAB-Tgm, alone or in combination, was performed excluding from the case series 3 CLN harboring metastasis from MTC and 3 CLN affected by extrathyroidal cancers. Specificity, sensitivity, PPV, NPV and accuracy are reported in Table 3.

**Table 3:** Diagnostic performance of the different tests employed in the diagnosis of suspicious metastatic cervical lymph nodes (CLN) from thyroid cancer patients

**Test**	**Sensitivity**	**Specificity**	**PPV**	**NPV**	**Accuracy**
***PTC + ATC CLN (n = 29)***					
FNAB-C	88.5 (23/26) [69.9-97.6]	N.D. (0/0) [N.D.]	100 (23/23) [85.2-100]	N.D. (0/3) [0-70.8]	88.5 (23/26) [69.9-97.6]
FNAB-Tgp	92.3 (24/26) [74.9-99.1]	N.D. (0/0) [N.D.]	100 (24/24) [85.8-100]	N.D. (0/2) [0-84.2]	92.3 (24/26) [74.9-99.1]
FNAB-Tgm	87.5 (21/24) [88.1-100]	N.D. (0/0) [N.D.]	100 (21/21) [84.0-100]	N.D. (0/3) [0-70.8]	87.5 (21/24) [88.1-100]
FNAB-Tgp + -Tgm	100 (29/29) [88.1-100]	N.D. (0/0) [N.D.]	100 (29/29) [88.1-100]	N.D. (0/0) [N.D.]	100 (29/29) [88.1-100]
FNAB-C + -Tgp	96.6 (28/29) [82.2-99.9]	N.D. (0/0) [N.D.]	100 (28/28) [87.7-100]	N.D. (0/1) [0-97.5]	96.6 (28/29) [82.2-99.9]
FNAB-C + -Tgm	92.9 (26/28) [76.5-99.1]	N.D. (0/0) [N.D.]	100 (26/26) [86.8-100]	N.D. (0/2) [0-84.2]	92.9 (26/28) [76.5-99.1]
FNAB-C + -Tgp + -Tgm	100 (29/29) [88.1-100]	N.D. (0/0) [N.D.]	100 (29/29) [88.1-100]	N.D. (0/0) [N.D.]	100 (29/29) [88.1-100]
***Only PTC CLN (n = 26)***					
FNAB-C	95.7 (22/23) [78.1-99.9]	N.D. (0/0) [N.D.]	100 (22/22) [84.6-100]	N.D. (0/1) [0-97.5]	95.7 (22/23) [78.1-99.9]
FNAB-Tgp	100 (23/23) [85.2-100]	N.D. (0/0) [N.D.]	100 (23/23) [85.2-100]	N.D. (0/0) [N.D.]	100 (23/23) [85.2-100]
FNAB-Tgm	90.5 (19/21) [69.6-98.8]	N.D. (0/0) [N.D.]	100 (19/19) [82.4-100]	N.D. (0/2) [0-84.2]	90.5 (19/21) [69.6-98.8]

Upper panel includes 26 CLN with metastatic PTC and ATC. Lower panel includes 23 CLN with only metastatic PTC. PPV, positive predictive value; NPV, negative predictive value; C, cytology; Tgp, thyroglobulin protein; Tgm, thyroglobulin mRNA. In bracket the number of cases. N.D., non-determined.

As it may be noticed, the diagnostic performances were comparable and not significantly different among FNAB-Tgp, FNAB-Tgm and FNAB-C. In addition, the combined use of FNAB-Tgp + FNAB-Tgm or FNAB-C + FNAB-Tgp + FNAB-Tgm did not improve significantly the diagnostic value of FNAB-C alone (Table 3).

When the analysis was restricted to the 26 CLN harboring metastatic PTC FNAB-C and FNAB-Tgp showed, respectively, 95.7 and 100% accuracy and, in the 20 CLN in which both diagnoses were available, a 95% overall agreement between the 2 tests was observed (Table 3). On the other hand, FNAB-Tgm showed 90.5% accuracy and an overall agreement of 94.7% (Cohen’s k = 0.64, p < 0.01) with FNAB-C in the 19 CLN in which both diagnoses were attained.

Regarding the 3 CLN harboring metastatic ATC (Table 1, CLN number 10, 12 and 13), FNAB-C identified correctly one of them (CLN number 10) and gave the erroneous diagnosis of metastatic PTC in the remaining two (from the same patient). As expected, in these 3 CLN FNAB-Tgp levels were low, only one (Table 1, CLN number 13) being above the cut-off value. On the other hand, FNAB-Tgm was positive in two CLN (Table 1, CLN number 10 and 12) and negative in one.

FNAB-Tgp provided a correct diagnosis in all 3 CLN with uninformative cytology. Also FNAB-Tgm provided correct diagnosis in 2 of the 3 CLN with inadequate cytology, being inadequate as well as in the third case.

## Discussion

Cytological evaluation of FNAB represents the gold standard technique for the diagnosis of CLN suspected to harbor metastatic disease from thyroid cancer as well as from other primary tumors. The technique accuracy, highly dependent on the experience and ability of the cytopathologist, has been reported to vary from 73% to 94% [17,18,22,25]. Over the last years, following clinical evidence showing that FNAB-Tgp in fine-needle washout improves the accuracy of FNAB-C in the evaluation of CLN metastases of DTC, it has been recommended the routine association of FNAB-Tgp with FNAB-C in the preoperative diagnosis of suspicious CLN [6,7,14-27,33]. We here report our experience with 35 CLN for which a definitive diagnosis was available, including cases harboring metastases from PTC, MTC, ATC and other primary tumors.

The results showed that, on 32 CLN harboring metastases from papillary and anaplastic thyroid carcinomas, FNAB-C had 88.5% accuracy, that is within the range reported in other studies [17,18,22,25]. When FNAB-Tgp and FNAB-Tgm were used singly they had a diagnostic performance comparable and not statistically different from that of FNAB-C. In addition, the combination of FNAB-Tgp and/or FNAB-Tgm with FNAB-C did not improve significantly the diagnostic value of FNAB-C. On the other hand, both FNAB-Tgp and FNAB-Tgm compared favorably with FNAB-C. As expected, a major disagreement among the three diagnostic tests was observed in the 3 CLN harboring metastasis from ATC (Table 2, CLN number 10, 12 and 13). It is worth to note that although ATC are undifferentiated cancers, i.e. devoid of thyroid specific gene expression, we consider the absence or the low level (below the cut-off value of 1 ng/FNAB) of Tgp or the absence of Tgm as false negative results. In fact, in one of the three metastatic CLN from ATC the detectable Tgp correctly diagnosed one of them (CLN number 13), and the detectable Tgm was diagnostic in the other two (CLN number 10 and 12).

A major diagnostic value of FNAB-Tgp and FNAB-Tgm was observed in cases of uninformative FNAB-C, given that in all 3 CLN characterized by inadequate cytology FNAB-Tgp provided the correct diagnosis. In these cases, FNAB-Tgm also provided the correct diagnosis in 2 of them, being inadequate in one. Other instances in which the combined use of FNAB-Tgp, FNAB-Tgm and FNAB-CTm is valuable include those cases in which FNAB-C diagnosis is not consistent with patient’s clinical and biochemical parameters. This was the case (Table 1, CLN number 19 and 20) of the patient harboring metastatic MTC and characterized by elevated level of serum calcitonin in which FNAB-C indicated metastatic PTC, while on the contrary both FNAB-Tgp and FNAB-Tgm were negative, and FNAB-CTm positive.

## Conclusions

**Figure 2:**
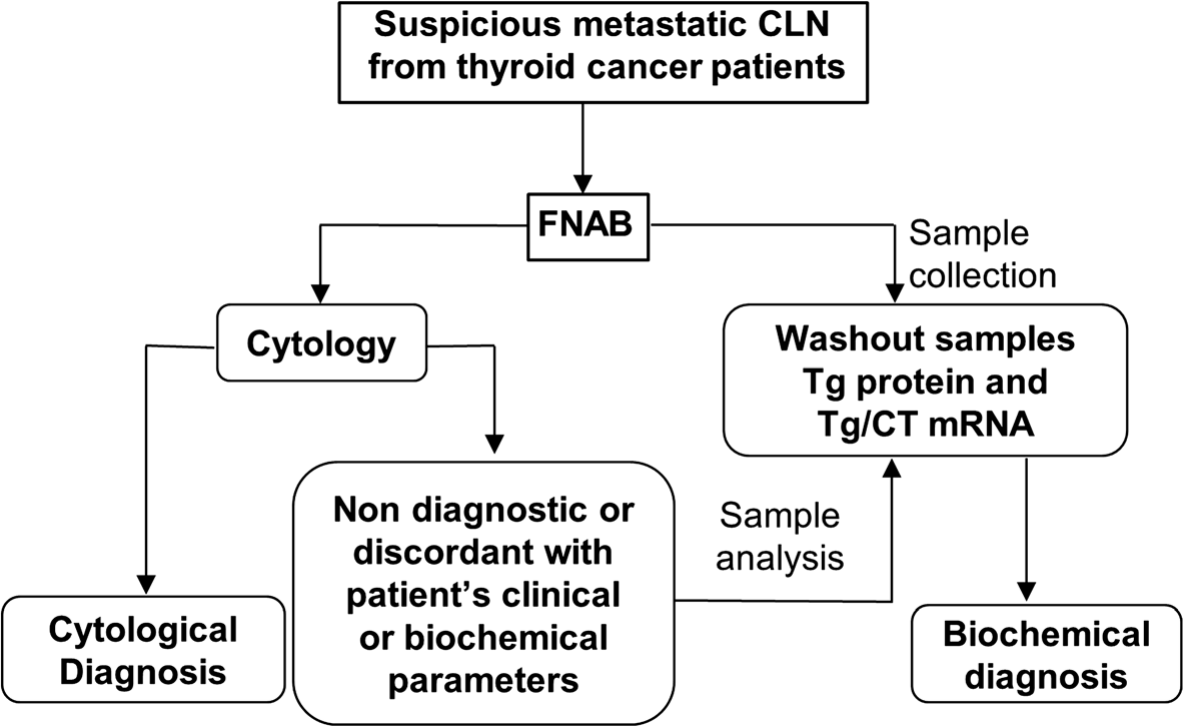
**Flow-chart for diagnosis of suspicious metastatic cervical lymph nodes (CLN) from thyroid cancer patients.** Following fine-needle aspiration biopsies (FNAB) samples for Tg protein and Tg/CT mRNA measurements should be collected but their analysis restricted to cases with uninformative or clinically unsound FNAB-C diagnosis. Tg, thyroglobulin; CT, calcitonin.

FNAB-C remains the gold standard technique for the diagnosis of suspicious CLN due to its ability to efficiently diagnose metastasis not only from thyroid cancers, but also from other primary tumors. If confirmed on larger case studies, the results of this study suggest that samples for Tg/CT mRNA and Tg protein measurements from CLN suspicious for metastatic thyroid cancer should be always collected (Figure 2), but their measurements should be restricted to cases in which FNAB-C give uninformative or inconsistent diagnosis with respect to patient’s biochemical and/or clinical parameters. Finally, it is also worth to mention that this approach may significantly reduce the costs of the management of patients with thyroid cancer whose incidence has been increasing over the last years, being actually the fifth most common cancer in women.

## Competing interests

The authors declare that they have no competing interests.

## Authors’ contribution

EB, SS, PM, EDeA and SU conceived the study. EB, CDG, AA, LG, GC and EDA run the experimental procedures and organized data collection. SU and CDV performed the statistical analysis. SS, PM, EDeA and SU drafted the manuscript. All authors read and approved the final manuscript.

## Pre-publication history

The pre-publication history for this paper can be accessed here:

http://www.biomedcentral.com/1472-6890/13/7/prepub
